# DISGROU: an algorithm for discontinuous subgroup discovery

**DOI:** 10.7717/peerj-cs.512

**Published:** 2021-04-27

**Authors:** Reynald Eugenie, Erick Stattner

**Affiliations:** 1 Laboratory of Mathematics, Computer Science and Applications, Université des Antilles, Pointe à Pitre, Guadeloupe, France; 2 Laboratory of Mathematics, Computer Science and Applications, Université des Antilles, Pointe a Pitre, Guadeloupe, France

**Keywords:** Data mining, Subgroup discovery, Descriptive modeling, Knowledge discovery

## Abstract

In this paper, we focus on the problem of the search for subgroups in numerical data. This approach aims to identify the subsets of objects, called subgroups, which exhibit interesting characteristics compared to the average, according to a quality measure calculated on a target variable. In this article, we present DISGROU, a new approach that identifies subgroups whose attribute intervals may be discontinuous. Unlike the main algorithms in the field, the originality of our proposal lies in the way it breaks down the intervals of the attributes during the subgroup research phase. The basic assumption of our approach is that the range of attributes defining the groups can be disjoint to improve the quality of the identified subgroups. Indeed the traditional methods in the field perform the subgroup search process only over continuous intervals, which results in the identification of subgroups defined over wider intervals thus containing some irrelevant objects that degrade the quality function. In this way, another advantage of our approach is that it does not require a prior discretization of the attributes, since it works directly on the numerical attributes. The efficiency of our proposal is first demonstrated by comparing the results with two algorithms that are references in the field and then by applying to a case study.

## Introduction

The field of *Data Science*, which aims to extract of knowledge from various kinds of data, has become a very active area of research in recent years. This strong enthusiasm for the discipline can be explained by the wide range of problems that can be addressed today: pattern search, cluster identification, automatic classification, extraction of frequent patterns, etc. Traditionally, data analysis approaches are separated into two families (Williams, 2011): (i) *predictive approaches* that exploit past data to make assumptions about the future and (ii) *descriptive approaches* that aim to highlight various kinds of models that summarize data and their underlying structure.

A prolific field on this domain is the pattern mining. Ventura et al. present pattern mining as a way to make sense of data which could be considered as messy (Ventura & Luna, 2018) and describe the main subcategories structuring the domain as follows: (i) Emerging Patterns, (ii) Class association rules, (iii) Exceptional models mining and (iv) Subgroup Discovery.

This paper focuses on the subgroup discovery problem (Atzmueller & Puppe, 2006). This approach aims to identify, from data, the subsets of objects, called *subgroups*, which exhibit interesting characteristics according to a quality measure calculated on a target label. The quality measure thus allows to compare the subgroups and to identify the one who maximizes it. This type of approach is used to highlight subsets of objects, hidden in data that exhibiting interesting characteristics deviating from the average.

Although the search for subgroups is a relatively new branch of the field of knowledge extraction from data, several approaches have already been proposed in the literature. While the first approaches of the domain carried out discretizations to realize classes on attributes (Atzmueller, 2015), some recent approaches have attempted to exploit numerical attributes directly with the aim to perform attribute ranges for identifying the most relevant intervals defining subgroups (Nguyen & Vreeken, 2016).

In this paper, we focus on the search for subgroups and we propose a new approach called *DISGROU* which identifies subgroups whose attribute intervals may be discontinuous. Unlike the main approaches in the field, the originality of our approach lies in the way it breaks down the intervals of the attributes during the subgroup research phase. Indeed the main approaches in the field identify groups only over continuous intervals that results in the identification of groups defined over wider intervals thus containing some irrelevant objects that degrade the quality function. The basic assumption of our approach is that the range of attributes defining the subgroups can be disjoint to improve the quality of the identified subgroups. Due to the process of finding discontinuous intervals introduced in DISGROU, additional operations are performed compared to the other approaches. More particularly, the construction of the FP-TREE, including multiple nodes defining selectors on the same attribute, induces an enlargement of the depth, which generates a greater cost on the calculation time on large datasets. However, another advantage of our approach is that it does not require a prior discretization of the attributes, since it works directly on the numerical attributes to perform the attribute splitting.

In this work, we detail the DISGROU algorithm we propose, which searches for subgroups on discontinuous intervals and which fully exploits the parallelization during the research phase of the subgroups to support the largest number of candidates generated due to our interval splitting approach. We demonstrate the efficiency of our approach by comparing the results with *SD-MAP* and *OSMIND* which today are the two reference algorithms in the field. Thus, by applying our approach to the benchmark of the four datasets traditionally used in the field, we show how the quality of the subgroups can be improved thanks to DISGROU. Finally, our approach is applied on the case study of the production of Banana in the Guadeloupe island.

The paper is organized as follows. “Related Work” reviews the main subgroup search approaches in the literature. “DISGROU Algorithm” details our methodology and presents DISGROU, the algorithm which implements our approach. “Experimental Results” is devoted to the performance of our approach, based on several experimental results comparing our approach to the 2 main approaches in the field. Performance is compared both quantitatively and qualitatively. In “Case Study: Banana Yield in the French West Indies” we apply the approach to the case study of agriculture in the French Caribbean island of Guadeloupe, for which we are particularly interested in studying the banana yield. Finally, “Conclusion” concludes and presents our future directions.

## Related Work

The pattern mining is an important subject in data mining. This field gathers many methods, such as (i) Emerging Patterns (García-Vico et al., 2016; Bayardo, Agrawal & Gunopulos, 2000), (ii) Class association rules (Ma, Liu & Hsu, 1998; Luna et al., 2015), (iii) Exceptional models mining (Leman, Feelders & Knobbe, 2008; Duivesteijn, Feelders & Knobbe, 2016) and (iv) Subgroup Discovery (Herrera et al., 2011; Helal et al., 2019). In this paper, we will focus on the latter.

Subgroup discovery is a descriptive data analysis technique in which the objective is to identify particular groups of transactions exhibiting good “quality” regarding a target attribute. The idea of a quality function was introduced in (Aumann & Lindell, 2003), in which Aumann and Lindell presented qualitative association rules where the behaviour (the right-hand side of the rule) was a measure to describe the distribution of the subset (the left hand of the rule). Herrera and al. (Herrera et al., 2011) pinpoint many quality measures which can serve as a foundation for quality function (measures on complexity, generality, precision and interest). Later, Atzmueller categorized the quality function in two groups (Objective and Subjective measures) (Atzmueller, 2015). In this paper, we will consider the quality function which was presented in their work for Numeric Target Quality Function:

(1) $$q_{a}(P)=n^{a}(m_{P}-m_{0})$$

where $q_{a}$ is the quality function, P the subgroup, n the number of elements in the subgroup, a∈[0;1] a parameter to adjust the weight of n in the final result, $m_{P}$ and $m_{0}$ respectively the means of the target value of the subgroup and the whole data set.

They also pinpoint three main categories of subgroups discovery algorithms which use those principles: **(i)** Extensions of classification algorithms, **(ii)** Evolutionary algorithms for extracting subgroups and **(iii)** Extensions of association algorithms.

Several approaches can be classified in the family of the *extensions of classification algorithms*, such as EXPLORA (Klösgen, 1996) and MIDOS (Wrobel, 1997), pioneers in the domain that uses decision trees which will be exhaustively walked through. Each of them show a particularity, as EXPLORA can also be used for heuristic subgroup discovery without pruning whereas MIDOS will use safe pruning and optimistic estimation on a multi-relational data base. We can also cite the algorithms proposed in (Klösgen & May, 2002; Gamberger & Lavrac, 2002; Lavrač et al., 2004; Lavrač, Železny & Flach, 2002) which essentially use the well-know Beam Search (Ney et al., 1987, 1992) approach as the search strategy.

On another hand, the family of *evolutionary algorithms* (Del Jesus et al., 2007; Berlanga et al., 2006; Carmona et al., 2010) have attempted to exploit the well-known concept of bio-inspired algorithms to extract subgroups from data. One of their common particularities lies in the fact that they use fuzzy rules based description language.

Nevertheless, one of the main drawbacks of these two families of algorithms lies in the fact that they mainly use nominal or categorical variable as their attribute target. However, in real datasets the target variable may often be a numerical value. In such a case, the solution for using the previous algorithms was to discretize the target variable. This implies a loss of information, which is why a current evolution of the Pattern Mining algorithms seeks to overcome this limitation (Luna et al., 2014; Proença et al., 2020).

In the case of the subgroup discovery algorithms, it is implemented through *association algorithms*. Indeed, they have the objective to exploits various kinds of target variables: binary variables (Grosskreutz et al., 2008; Atzmueller & Puppe, 2006), categorical variables (Kavšek, Lavrač & Jovanoski, 2003; Mueller et al., 2009; Grosskreutz et al., 2008; Atzmueller & Puppe, 2006) and also numerical variables (Atzmueller & Puppe, 2006; Millot, Cazabet & Boulicaut, 2020). In this last category, Atzmueller and al. showed excellent results in (Atzmueller & Puppe, 2006) with the SD-MAP algorithm. Although this method needs a discretization on the non-target attributes, for years it was the reference in the domain. Very recently, Millot et al. have proposed the OSMIND algorithm (Millot, Cazabet & Boulicaut, 2020), which performs even better score than SD-Map. Their method consists in a fast but exhaustive search without prior discretizations of the data, resulting in an optimal subgroup.

Nevertheless, the multiple methods previously described only consider simple continuous intervals in the subgroup description. However, the possibility of identifying subgroups on discontinuous intervals would allow to have a more precise description of the subgroup and to increase its quality. For instance, let’s consider a dataset with an attribute $att_{i}$. Even if good transactions lies in an interval [a,b], a part of this interval $\left[a^{\prime },b^{\prime }\right]\subset \left[a,b\right]$ may display very low quality transaction, reducing the overall quality. Thus the objective of our approach aims to filter the low quality part and return the optimal interval that may be disjoint such as $\left[a,a^{\prime }\right]\cup \left[b,b^{\prime }\right]$ intervals. In this paper we present DISGROU, the first approach that extracts subgroups with discontinuous intervals in their subgroup description in order to highlight meaningful subgroups filtered from low quality parts.

## DISGROU Algorithm

The main idea of the approach we propose is to identify, for each attribute, the ranges of values that must characterize the subgroups. Consequently, unlike the main approaches of the domain that only look at continuous intervals, the algorithm DISGROU searches for subgroups that can involve discontinuous intervals through possible unions on attributes.

**Figure 1:**
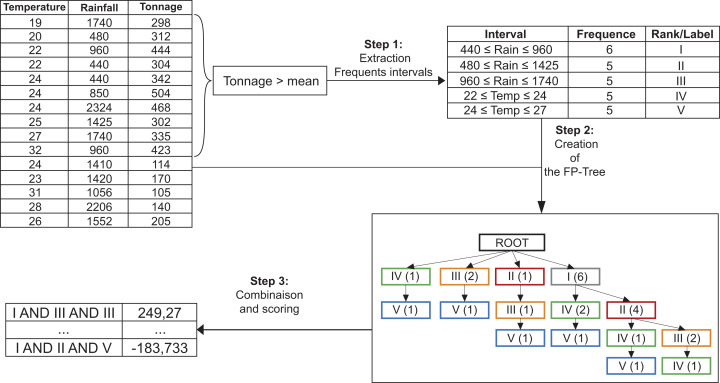
The three main steps of the DISGROU algorithm.

More precisely, in the Algorithm 1, DISGROU performs this task in 3 main steps, as depicted on Fig. 1.

1. **Extraction of the raw selectors**, the first intervals which will serve as a basis for the composition of the subgroups (detailed in Algorithm 2).
2. **Construction of the FP-Tree** by using the extracted selectors (see Algorithm 3).
3. **Combination of the nodes of the FP-Tree** to create the subgroups. Their score will be determined at this point, and the bests will be conserved (as shown is Algorithm 4).

**Algorithm 1:** DISGROU.

**Require:** *A* : list of attributes, *A_target_* : target variable, *T* : list of transactions , β : support threshold
**Ensure:** *listSubGroup* : list of the subgroup sorted by their quality
1: *S* : set of selectors
2: *Tree* : FP-Tree
3: *listSubGroup*: list of the identified subgroup
4: *S* = *MAKE_RAW_SELECTORS*(*A*, *A_target_*, *T*)
5: *sort*(*S*)
6: *Tree* = *MAKE_TREE*(*T*, *S*)
7: *listSubGroup* = *BRANCH_COMBINER*(*FPTree*)
8: *getBestScore*(*listSubGroup*)

**Algorithm 2:** MAKE RAW SELECTOR.

**Require:** *A* : list of attributes, *A_target_* : target variable, *T* : list of transactions , β : support threshold
**Ensure:** *S* : List of the selectors s for which *ext*(*s*) ∩ *pos*(*T*) > β
1: *S* = {}
2: **for** *i* **from** 0 **to** |*A*| **do**
3: *interval* = {*min_A_i__*; *max_A_i__*}
4: *add*(*interval*, *S*)
5: *addSubIntervalWithErosion*(*A_i_*, *A_target_*, *pos*(*T*), *interval*, β)
6: **end for**
7: **return** S

**Algorithm 3:** MAKE TREE.

**Require:** *T* : list of transactions, *S* : list of selectors
**Ensure:** *FPTree* : partial FP-Tree of the thread
1: *FPTree* = *newRootTree*()
2: **for all** t ∈ *T* **do**
3: *actNode* = *root*(*FPTree*)
4: **for all** *s* ∈ *S* **do**
5: **if** *valid*(*t*, *s*) **then**
6: **if** *s* ∈ *children*(*actNode*) **then**
7: *actNode* = *child*(*actNode*, *s*)
8: *addToNode*(*t*,*actNode*)
9: **else**
10: *childNode* = *newNode*(*t*, *s*,*actNode*)
11: *actNode* = *childNode*
12: **end if**
13: **end if**
14: **end for**
15: **end for**
16: **return** *FPTree*

**Algorithm 4:** BRANCH COMBINER.

**Require:** *FPTree* : the generated FP-Tree, *listSelector* : list of the selectors
**Ensure:** *listSubGroup* : List of the candidate subgroups with their quality
1: *listSubGroup* = {}
2: **for all** *interval_i_* ∈ *listSelector* **do**
3: *newSet* = *allBranchesWith*(*intervali*)
4: *removeNonFrequent*(*newSet*)
5: **for all** *subGroups* ∈ *combinaisons*(*newSet*) **do**
6: *add*(*s*, *listSubGroup*)
7: **end for**
8: **end for**
9: **return** *listSubGroup*

Furthermore, two particularities can be observed in our method. Many selectors on the same attribute may appear on the same branch as on the example in Fig. 1 with the leftmost branch for instance. This particularity induces a special treatment that will be detailed further. Also, the selectors can either be continuous intervals, or union of two intervals. This second particularity will allow us to extract finer subgroups descriptions.

An example can be taken on the banana crop : In (Ganry, 1973), Ganry states that the growth temperature of the banana crop is defined between 9 °C and 40 °C, and that the optimal temperature should be around 28 °C. As an example, let’s consider a range of five degrees around this optimal value as the optimal interval. The interval [23;33] will be considered as the interval of temperature for a good harvest. However in some region a fungus named black sigatoka parasite the leaves of the banana tree. From the infection result harmful effect on the growth of the crops and on the overall productivity. In (Jacome, 1992), Jacome defines the optimal temperature of this fungus is in the range of [25;28] degree. The existence of this parasite drops the value of the [23;33] interval. With our methods, we should be able to remove [25;28], and extract the optimal [23;25]∪[28;33] intervals.

More precisely, DISGROU performs as follows: let D be the dataset constituted with a set of attributes A, $A_{target}$ the target variable on which the quality of the subgroup will be calculated and T a set of transactions.

In this paper, the study mainly focuses on continuous target variable, but it can also be applied to discrete variable. Indeed, in the case of discrete numerical target, the dataset can be used without prior modification using the mean of the target. Otherwise, in the case of nominal variable, the target can be converted in numerical values in order to be used as discrete numerical variables.

For each attribute $A_{i}$ in A, the values $Min_{i}$ and $Max_{i}$ are defined respectively as the minimal and maximal value of the dataset on the attribute $A_{i}$ (Algorithm 2, line 3).

The tasks of the DISGROU algorithm can be divided in three main steps as shown on Algorithm 1: (i) The creation of the raw selectors for each attribute (line 4), (ii) the creation of the FP-Tree using those selectors (line 6) and finally (iii) the combination and scoring of the branches of the tree from which result the final subgroups (line 7).

The objective of the first step is to extract raw selectors which will be used in the FP-Tree (see Algorithm 2). A selectors s is defined with a couple of objects (lh(s),rh(s)), with (i) lh(s) its left hand, an element $A_{i}$ of A and (ii) rh(s) the right hand, an interval or a couple of disjoint intervals on the attribute, forming a subset of $\left[Min_{i};Max_{i}\right]$. Moreover for each selector s, its extent ext(s) can be defined as set of transactions such as $\forall t\in ext(s),val(t,A_{i})\in rh(s)$, with $val(t,A_{i})$ returning the value of the transaction t on the attribute $A_{i}$.

In order to extract the raw selectors S, DISGROU bases its treatment on pos(T), the subset of transactions with a higher value on their target variable than the mean of the population. Then DISGROU starts with the complete interval of each attribute $A_{i}$ and erodes them into smaller sets in the method *addSubIntervalWithErosion* (line 5). Each set extracted have to verify the following rules:

1. The subset can be defined by either a continuous interval or the union of two intervals [a;b]∪[c;d] such as [a;b]∩[c;d]={∅}.
2. The number of transactions of [a;b] has to be higher than a fifth of the number of elements in [c;d] and conversely, in order to mitigate the union between a meaningful interval and an irrelevant one.
3. The number of transactions of the subset have to be higher than a given support threshold β.

At the end of this first step, the algorithm creates for each attribute *A_i_* a set *S_i_* of selectors *s_i_*_, *j*_ which are merged in *S*.

In the second part, DISGROU uses the selectors extracted in step 1 in order to build the FP-Tree. This step is detailed in Algorithm 3. Each node of the tree represents a triplet build with a selector, the number of transactions and the sum of the value in the target variable for the transactions which had reached the node. In order to obtain the final tree, the algorithm classifies all of the selectors by their frequencies.

For each transaction of the database, the FP-Tree is modified as follows: The process starts at the root and check if the transaction t is included in the first selector $s_{1}$. While it’s not included, DISGROU recursively move to the next selector $s_{i+1}$. When t matches $s_{i}$, if the current node has a child which corresponds with $s_{i}$, the value of the child is incremented. Otherwise, the algorithm creates the child node. Then, the child becomes the current node, and DISGROU continue to browse the selectors until the last one.

At last, the third part of the algorithm is dedicated to the scoring of the possible subgroups. The Algorithm 4 takes all of the combinations of the selectors on the branches with the corresponding score, then merge the count of the transactions and the sum of the target variable with elements constituted by the same selectors on other branches. Then, for each combination of selector DISGROU uses the aggregated value and generates the corresponding score. Finally, the subgroups with the best quality according to the quality function in Eq. (1) are returned as the result of the algorithm.

The DISGROU algorithm has been implemented in *Java* and it is available on GitHub (http://pcaltay.cs.bilkent.edu.tr/DataSets/).

## Experimental Results

This section focuses on the performances of the DISGROU algorithm we propose. We compare the results with two reference algorithms, SD-Map (Atzmueller & Puppe, 2006) and OSMIND (Millot, Cazabet & Boulicaut, 2020).

Some algorithms like SD-Map may be more efficient with discrete variables, but it highlights a major issue, which is the identification of the optimal discretization for the non-target attributes: type of discretization, number of classes, sizes of classes, etc. That is why other algorithms such as OSMIND or DISGROU propose a way to overcome this limitation by addressing raw data directly. In this paper, the data was used without prior discretization, which is the first kind approach when we don’t have any a priori on the dataset.

In this experiment, we apply the algorithms on a benchmark of four algorithms traditionally used in the field. The results are analysed from (i) a quantitative point of view through which the quality values are compared for each algorithm, and (ii) a qualitative point of view through which the extracted subgroups are compared for each algorithm.

### Test environment

The four datasets which were used come from the Bilkent repository (http://www.fao.org/faostat/en/#data/QC) traditionally used as a benchmark for evaluated performances of subgroup discovery algorithms.

1. Airport (AP), which contains air hubs in the United States as defined by the Federal Aviation Administration.
2. Bolt (BL), which gathers data from an experiment on the effects of machine adjustments on the time to count bolt.
3. Body data (Body) which represents data on body Temperature and Heart Rate.
4. Pollution (Pol) which are data on pollution of cities.

For each dataset, all attributes are numeric attributes that have not been discretized beforehand. Such a comparison is interesting as the discretization may not be an intuitive operation for a complex dataset, due to the multiple existing ways. Thus observing these results under those circumstances may reveal the actual capacity of the subgroup discovery algorithm in front of this configuration. On a first hand, we will compare the score of the best subgroup extracted by each algorithm on each dataset. After that, we focus our interest on the evolution of the scoring by varying the size of the datasets. Finally, we will compare the means of the score from one to ten best subgroup for each algorithm. In our experiments, we always use the lowest β threshold with DISGROU that gives the best results.

### Quality of the best subgroup

**Figure 2:**
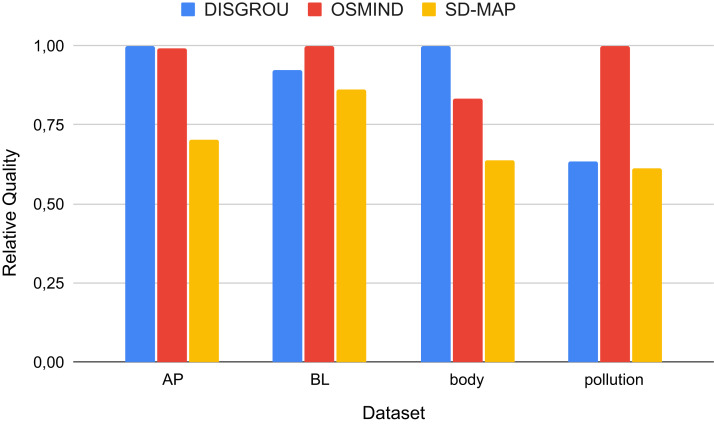
Comparison of the quality of the best subgroups identified normalized by the best score.

In a first step, we have compared the quality value returned for the best subgroup identified by each algorithm on the 4 datasets. For each dataset, the quality value has been normalized between 0 and 1 and is presented on Fig. 2.

Firstly, we can observe that DISGROU always provides better results than SD-MAP. Moreover, on the 4 datasets used, DISGROU gives better results than OSMIND on 2 of them (AP and body). This demonstrates the interest of our approach since it allows to identify better subgroups on certain datasets.

In more details, the results are very closed for the three algorithms on dataset BL. This suggests that the specific interval splitting introduced in DISGROU does not improve results on this dataset since the subgroups identified by all algorithms are pretty much the same.

Finally, the worst results are observed on dataset Pollution. This can be explained by the number of attributes on this dataset for which the depth of the FP-Tree tends to greatly increase. Indeed, the β threshold has to be set at a high value in order to diminish the number of selectors, and thus the overall quality of the subgroups greatly decrease.

These results demonstrate the good performance of the approach we propose. On average, the gain on the quality value of the best subgroup identified is 18.58% compared to SD-MAP, but −6.64% compared to OSMIND. The loss regarding OSMIND in mainly due to the pollution dataset on which the current version of our approach does not extract a great subgroup, but without this specific case, DISGROU bring a gain of 9.98% in comparison to OSMIND.

**Figure 3:**
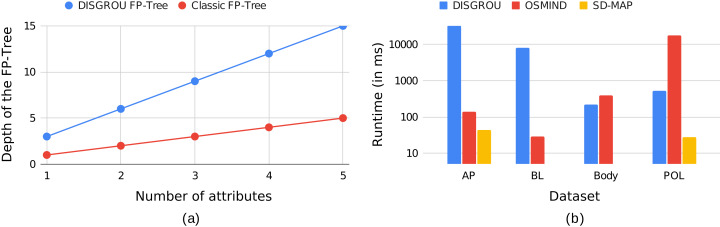
Example of the evolution of (A) the depth of a FP-Tree with three selectors by attributes, when the number of attributes varies and (B) the runtime of the algorithms on the different dataset.

However, it is important to note that the FP-Tree used in DISGROU is special. In traditional approaches, the depth of the tree is bounded by the number of attributes. Indeed, intersections of selectors on a same attribute are often empty, limiting the number of elements per branches. In the FP-Tree introduced in DISGROU the final selectors of each attribute are created throw a combination of raw selectors with many intersections. Thus, on a same branch, a large number of selectors may exist, increasing the depth of the tree, as depicted in Fig. 3A.

This figure represents the evolution of the depth of a FP-Tree in regard to the number of attributes. In this example, each attribute generates three selectors. In this figure, we can see that the depth of the tree is multiplied by the number of selectors per attributes. More generally, the depth of a classic FP-Tree is limited by the number of attributes, while the depth of the FP-Tree in DISGROU is limited by the number of raw selectors, inducing a greater cost on the calculation time on large datasets. The observation can be supplemented by the Fig. 3B. DISGROU was fixed with a threshold β>80%, and tend to be more time consuming than the other algorithms, especially SD-MAP. In the “Conclusion”, propositions to overcome this challenge will be discussed about.

### Subgroup quality and dataset size

**Figure 4:**
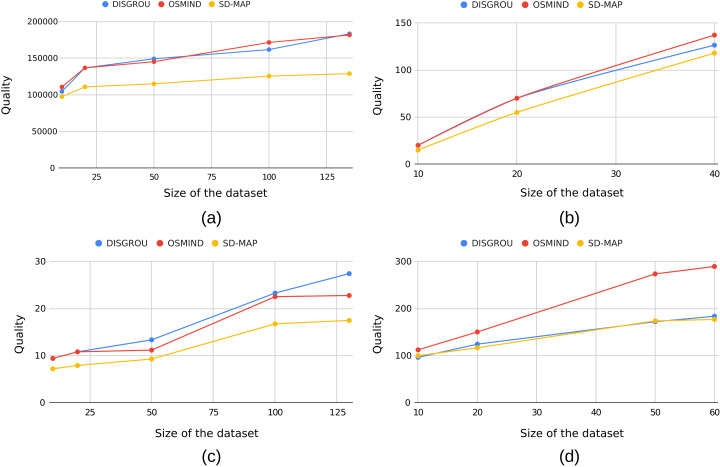
Evolution of quality of the best subgroup according to the dataset size with (A) airport, (B) bolt, (C) body and (D) pollution.

In a second step, we have compared the performance of the algorithms with different dataset sizes. The size of sub-datasets varies by selecting respectively 10, 20, 50 and 100 elements according to the data as well as their maximal size. The Fig. 4 shows the evolution of the scoring of the best subgroup according to the number of transactions.

First of all, we can observe that the trends remain globally stable over all datasets.

OSMIND and DISGROU provide the best quality values on AP, BL and Body. Although the values are relatively close, it is still interesting to observe that in several configurations, the DISGROU algorithm identifies subgroups with better quality values than OSMIND. This is, for example, the case on the Body dataset from 50 transactions or on the AP dataset with 50 and 125 transactions. This demonstrates, once again, the value of the approach we are proposing.

Also, if we focus on the Body dataset (see Fig. 4C), the tail of the curve differs significantly, as the SD-Map algorithm and the OSMIND seems to slow their increase, when the DISGROU seems to maintain its.

### Top best subgroups

Traditionally, subgroup research approaches focus only on the best subgroup as we have done in the “Quality of the Best Subgroup” and “Subgroup Quality and Dataset Size”. However, in a real-world context and particularly in a decision support context, it may be useful to be able to compare all the best subgroups.

**Figure 5:**
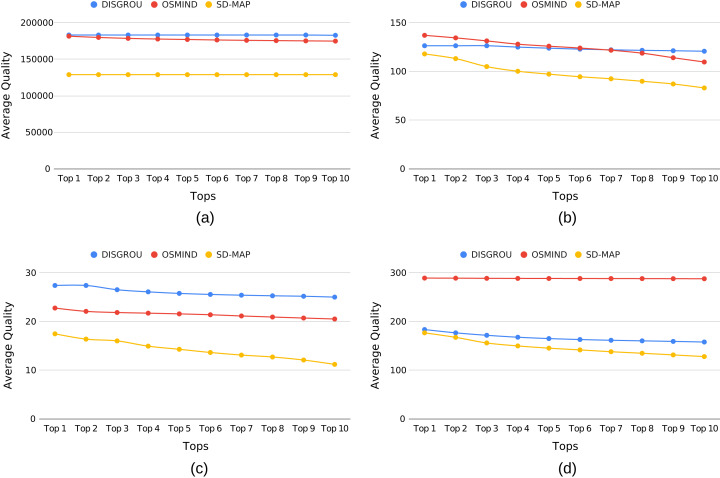
Evolution of the mean quality of the top best subgroups in regard of the number of subgroup with (A) airport, (B) bolt, (C) body and (D) pollution.

In this part of our experiments, we take another point of view by focusing on the 10 best subgroups extracted by the three algorithms. Figure 5 shows the average quality value from Top 1 to Top 10 best subgroups.

As previously observed, for AP and Body datasets, we can see that DISGROU provides best subgroups. When considering tops best (see Figs. 5A and 5C), it seems even more significant as neither OSMIND nor SD-Map draw closer to the quality of DISGROU. This suggests that our approach not only identifies the best subgroups on AP and Body, its also provide the numerous of subgroups close to the best.

A more significant change happens in Fig. 5B. Indeed whereas OSMIND gave a better result for the score of the best subgroup, at the top $7^{th}$ the trend is reversed. When the top $10^{th}$ is reached, the lead of DISGROU is even more visible.

The results observed on the Pollution dataset are consistent with what we observed previously.

### Structure of subgroups

In the last part of the experiments, we will focus on the subgroup structure extracted by each algorithm. One of the innovations introduced in the DISGROU algorithm is its capacity to extract subgroups with discontinued intervals for the attributes. In this section, we compared the structure of the extracted subgroup description by our approach with that extracted by the other two approaches.

Tables 1 to 3 show the extracted subgroups for Bolt, Airport and Body respectively. The Pollution dataset was discarded due to the very large number of attributes that does not easily allow the description of subgroups on a table. On these tables, each column corresponds to an attribute of the dataset and the value represents the interval identified on the subgroup. An empty cell means that this attribute does not participate in the definition of the subgroup.

**Table 1:** Subgroups description extracted on the Bolt dataset for each method.

Bolt							
Algorithm	Col0	Col1	Col2	Col3	Col4	Col5	Col6
SD-MAP (Score : 0.86)		[6;6]	[30;30]				
OSMIND (Score : 1.0)	[6;39]	[6;6]					[28.89;134.01]
DISGROU (Score : 0.92)	[6;8]∪[10;39]					[0;6]	

An interesting point can be pinpoint on subgroups identified on the Bolt dataset (See Table 1). Even though the quality of the subgroups of our algorithm is slightly lower than OSMIND in the Bolt dataset, the subgroups extracted by DISGROU only use two attributes when OSMIND used three, resulting is a more complex rule. Thus, even in cases where OSMIND shows better result, it may be easier to understand or exploit the subgroup description proposed by DISGROU.

This particularity is more visible in the Pollution dataset. Even through there is a drop in the quality of the extracted subgroup, OSMIND used 14 of the 15 attributes, resulting in a very complex and specific subgroup. On the other hand, DISGROU only use one attribute.

Regarding the subgroups identified on the Airport dataset (see Table 2), on top of DISGROU performed better than the other algorithms, the most interesting parts are the fact that it still uses less attributes, and the particular description of the subgroup. Some attribute seems more relevant on their excluded parts which led to the discontinuous interval. This relevance can be highlighted with DISGROU.

**Table 2:** Subgroups description extracted on the Airport dataset for each method.

Airport				
Algorithm	Sch_Depart	Perf_Depart	Enp_Pass	Freight
SD-MAP (Score : 0.70)				[300463.8;300463.8]
OSMIND (Score : 0.99)	[35891,322430]	[35273,332338]	[1362282,25636383]	[142660.95,352823.5]
DISGROU (Score : 1)	[134929;322430] ∪ [73300;92659]		[49572.7;352823.5] ∪ [5701.22;18041.4]	[2312455;7677769] ∪ [9332091;25636383]

The observations are different for the subgroups extracted on the Body dataset (see Table 3) for which DISGROU provides the best results. Indeed, we can see a strong similarity between the two subgroups extracted by SD-MAP and OSMIND. The right part of the DISGROU subgroup on body_temp is almost identical to the interval extracted by OSMIND. However, by adding the transactions which match the [97.8;98.0] intervals for the body temperature attribute, the quality of the corresponding subgroup increase. From a different point of view, [97.8;98.7] seems to be a good interval on the body_temp attribute, but the results are degraded by the part in ]98.0;98.3[, explaining the fact that OSMIND decided to only keep the left part.

**Table 3:** Subgroups description extracted on the Body dataset for each method.

Body Temperature—Heart Rate		
Algorithm	body_temp	gender
SD-MAP (Score : 0.63)	[98.6;98.6]	[2;2]
OSMIND (Score : 0.83)	[98.3,98.6]	[2;2]
DISGROU (Score : 1.0)	[97.8;98.0] ∪ [98.3;98.7]	[2;2]

## Case Study: Banana Yield in the French West Indies

**Figure 6:**
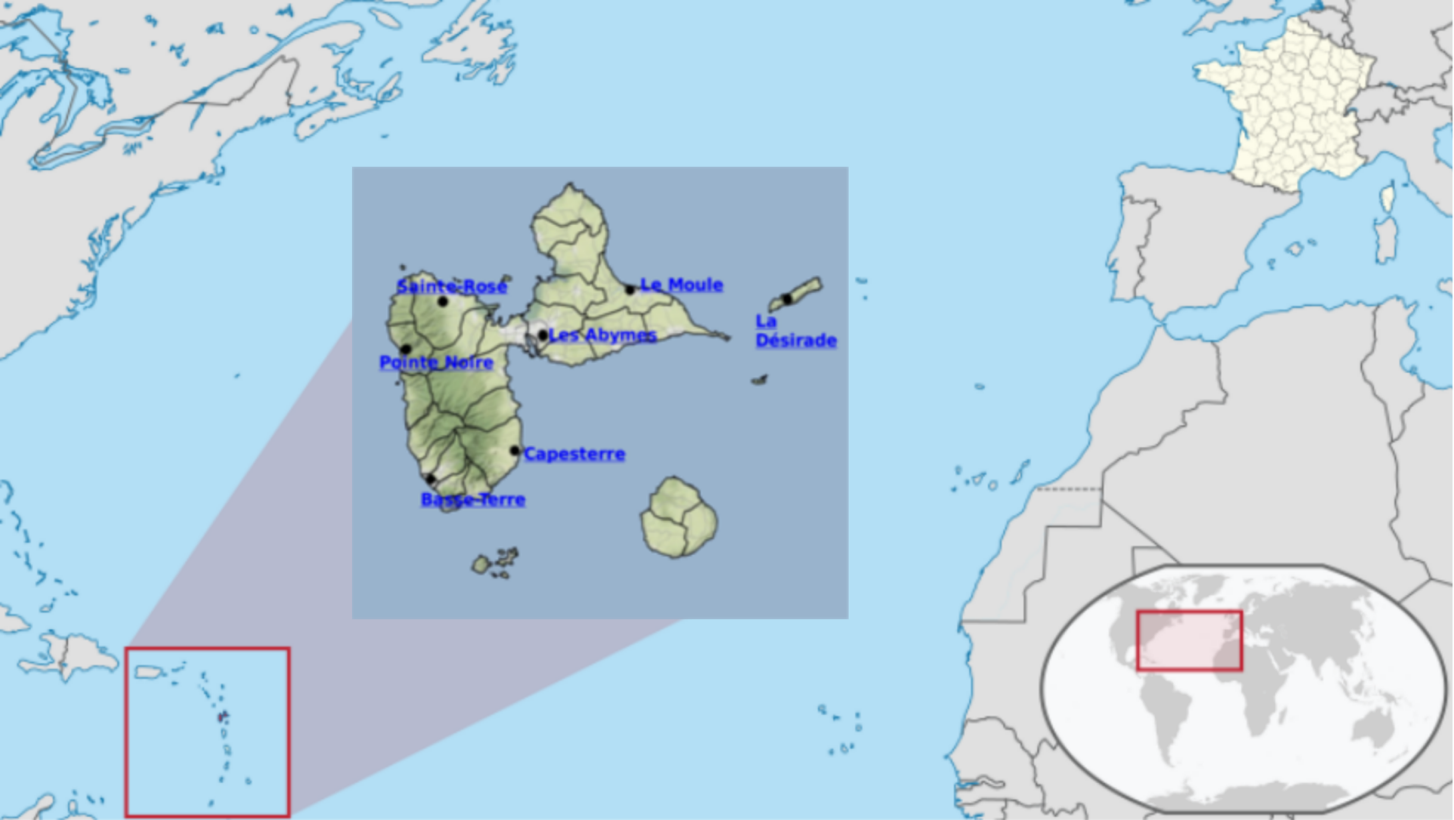
Guadeloupe island in the French West Indies. Source: https://upload.wikimedia.org/wikipedia/commons/7/77/Guadeloupe_in_France.svg.

In the last part of the work, we have applied the approach to a real case study: the yield of the banana crop in Guadeloupe, a little island in the French West Indies shown on Fig. 6.

For this study, we have used French weather sensors located in 7 cities of Guadeloupe (Le Moule, Les Abymes, Sainte-Rose, Pointe-Noire, Basse-Terre, Capesterre, La Désirade). Each sensor collects Temperature and Rainfall data since 1963 for the oldest. Data have been averaged in order to have the weather trends on the whole territory. Finally, the dataset has been supplemented with banana yields in the territory using data coming from the *Food and Agriculture Organization Corporate Statistical Database* (http://www.fao.org/faostat/en/\#data/QC).

Thus the dataset used contained 54 lines, each representing one year from 1964 to 2017. For each year, the dataset contains 3 attributes representing: (i) the average temperature, (ii) the average rainfall and (iii) the banana yield. The objective was to identify, using the subgroup discovery techniques, the climatic conditions, in terms of temperature and rainfall, for which the yield deviates significantly from the average yield. This approach is interesting because the banana is a crop that is very sensitive to climatic variation. In this context, the use of subgroups discovery methods offer good prospects to highlight climatic conditions that favour yields.

Table 4 presents the subgroups related to Banana yield extracted by SD-MAP, OSMIND and DISGROU.

**Table 4:** Subgroups description related to Banana yield for each method without discretization on the target variable.

Algorithm	Temperature	Rainfall	Scoring
SD-MAP		[4.53;4.53]	90,680.185
OSMIND	[24.55,25.65]	[3.23,4.14]	115,118.71
DISGROU	[24.4;24.8] ∪ [25.45;26.0]	[3.23;4.14] ∪ [4.95;6.14]	137,721.89

As expected, the first obvious difference is the ability of DISGROU to identify discontinuous subgroups. Indeed, the subgroups highlighted by the other approaches only involve continuous temperature and rainfall intervals. On this dataset, this specific subgroup research method introduce in DISGROU is relevant since the gain is 19% compared to OSMIND and 52% compared to SD-MAP.

In the Table 5, the same experiment was done while discretizing the target variable in four levels : **very low**([100000;150000[), **low** ([150000;200000[), **good** ([200000;250000[) and **very good** ([250000;300000[). Thus each transaction was assigned to the value in the middle of its class.

**Table 5:** Subgroups description related to normalized Banana yield for each method with discretization on the target variable in 4 classes.

Algorithm	Temperature	Rainfall	Scoring
SD-MAP		[3.23;3.23]	71,296.29
OSMIND	[24.5;26.0]	[2.72;4.14]	115,858.52
DISGROU	[24.4;24.55] ∪ [25.55;26.0]	[3.19;4.10] ∪ [5.25;5.34]	133,814.71

As a result, the trend observed in Table 4 is also observed with the discretized variable. The method is also effective on datasets with discretized target variable when it is converted by meaningful numerical values.

Obviously, an in-depth study should be validated by an agricultural specialist since many other factors can explain differences in yield, such as evolution in agricultural practices, changes in species or the use of pesticides. Nevertheless, the approach we propose could be relevant for specialists in the field agriculture since it is able to highlight more precise intervals of attributes involved in the subgroups. In a context of decision support, for example, the subgroups extracted by DISGROU could be used to adapt agricultural practices and improve the yields.

## Conclusion

In this paper, we have addressed the problem of the search for subgroups in numerical data. Unlike the main approaches in the field that extract subgroups on continuous intervals of attributes, the originality of DISGROU, the approach we propose, lies in its ability to identify subgroups on discontinuous intervals. Our contributions can be summarized as follows.

1. We have proposed a new algorithm which the search process, which recursively erodes the attribute values, allows the identification of subgroups that can be defined by interval unions. In addition, the algorithm performs this research process by parallelizing the calculations.
2. We have conducted experiments to compare DISGROU to the two reference algorithms in the field. The results of our experimentation highlighted the interest that lies in the use of discontinuous intervals in subgroup discovery. Indeed, we have shown the direct impact of the approach on the structure of the subgroups identified as well as the improvement in quality that is induced in some cases. Thus the proposed algorithm is able to extract these types of subgroups and can even compete with the reference in the domain.
3. Finally, we have applied the approach to the case study of the Banana yield on the Guadeloupe island in the French West Indies. In this case study the ability of the approach to extract much more precise subgroups has been demonstrated, which could prove useful in a decision support context.

Its result is an algorithm able to extract particular patterns through an adaptation of the FP-Tree. We have shown that DISGROU can even extract subgroups with better score than the best algorithms nowadays.

As perspective, we plan to address the scaling up of the approach and particularly the management of datasets with a large number of attributes.

As highlighted in our results, the main limits of DISGROU lies in the depth of the FP-Tree, which is expanded due to the use of many selectors from the same attribute on the branches. This particularity, with a naive walk through the tree increases the complexity of the algorithms which lead to a great loss in time.

Filtering the selectors at the moment of their creation to limit the number of overlapping ones may be a great track to diminish the depth of the FP-Tree. Another solution could be to investigate in a way to level the tree by reducing its depth to the detriment of its width. In the short term, we also want to introduce various pruning methods to improve the process.

Another interesting track which has to be studied is the meaning of the extracted subgroup. For now, we only focused on subgroup definitions with a maximum of two parts for each attribute, while keeping a balance between them. The meaning of the discontinuity, as well as the balance between each part deserves to be focused on in order to provide fully usable and meaningful subgroups.

## Supplemental Information

10.7717/peerj-cs.512/supp-1 Supplemental Information 1 DISGROU Source code.

## Additional Information and Declarations

### Data Availability

The following information was supplied regarding data availability: The datasets used for performance evaluation are available at the Function Approximation Repository: http://pcaltay.cs.bilkent.edu.tr/DataSets/. Data used: - Airport - Bolt - Body temperature and - Pollution The dataset used for the case study is available at the FAO: http://www.fao.org/faostat/en/\#data/QC. Country: Guadeloupe Elements: Yield Items: Bananas Year: from 1964 to 2017 The source code is available at GitHub: https://github.com/rey-sama/DISGROU.git.
